# Social context modulates digestive efficiency in greylag geese (*Anser anser*)

**DOI:** 10.1038/s41598-018-34337-3

**Published:** 2018-11-07

**Authors:** Didone Frigerio, Kurt Kotrschal, Carla Fabro, Verena Puehringer-Sturmayr, Lara Iaiza, Josef Hemetsberger, Federico Mason, Chiara Sarnataro, Stefano Filacorda

**Affiliations:** 1 Core Facility Konrad Lorenz Forschungsstelle for Behaviour and Cognition, University of Vienna, Fischerau 11, A-4645 Gruenau im Almtal, Austria; 2 Department of Behavioural Biology, University of Vienna, Althanstrasse 14, A-1090 Vienna, Austria; 3 Department of Agricultural, Food, Environmental and Animal Sciences, University of Udine, Udine, Italy; 4 Institute of Animal Reproduction and Food Research of the Polish Academy of Sciences, 10–748 Olsztyn, Poland

## Abstract

In group-living animals, social context is known to modulate physiology, behaviour and reproductive output as well as foraging and nutritional strategies. Here we investigate the digestive efficiency of 38 individuals belonging to different social categories of a semi-feral and individually marked flock of greylag geese (*Anser anser*). During 9 consecutive days in winter 2017, when the ground was fully covered with snow (i.e. no grass or other natural forage available) and the accessible food was standardized, 184 individual droppings were collected and analysed to estimate the apparent digestibility of organic matter (ADOM). Lignin was used as an indigestible internal marker in the food and droppings. The digestive efficiency was higher in pairs with offspring as compared to pairs without offspring or unpaired birds. Furthermore, individuals with high ADOM were more likely to breed successfully in the following season than those with low ADOM. Our findings demonstrate that social status modulates digestive efficiency, probably via a chain of physiological mechanisms including a dampened stress response in individuals enjoying stable social relationships with and social support by their family members (i.e. their own pair-partner and offspring). Our findings underline the importance of the social network in modulating physiology, such as digestive efficiency, and ultimately reproductive success.

## Introduction

In group-living animals, social context is known to modulate physiology, behaviour and reproductive output^1–5^. The social status of an individual also affects its access to resources^6,7^, and social interactions can have additional effects on energetic gain or predation risk^8,9^.

Competitive and agonistic interactions, for instance, can constrain the coping capacity of low-ranking individuals^10,11^, whereas social instability is generally considered a potent stressor in social animals^12^. One key mechanism to alleviate social stress is emotional social support, defined as the stress-reducing effect of the presence of a social partner^13^. In fact, social buffering of both the hypothalamic-pituitary and the sympathetic-adrenergic stress axis is a well-known mechanism in mammals^14^ and birds^15–18^. Individual foraging behaviour and food uptake is also affected by social position. Consequently, dominant individuals generally profit from earlier access to food sources, whereas low-ranking individuals may be constrained in this respect^19,20^. Furthermore, even the gut microbiome, crucial in the regulation of behaviour^21^, is affected by individual stress coping^22,23^. For instance, in baboons (*Papio* spp.) grooming partners had similar gut microbiotas^24^. In black howler monkeys (*Alouatta pigra*) as well, individuals spending time in contact and/or in close proximity with each other had similar gut microbial communities^25^. The gut microbiota is also known to modulate the hormonal stress response and thereby affect behaviour^26,27^. Moreover, postnatal microbial colonization could affect the development of brain plasticity in early stages of life and therefore, over the long-term, physiological responses. For instance, in rats the absence of a gut microbiota increased the hormonal and behavioural responses to acute stress situations^26,27^. The interplay between these complex mechanisms is termed the brain-gut signalling system, which is related to both short- and long-term stress and health^28^.

Most goose species live in large flocks with a complex social structure, as there is evidence for extended family bonds and female-centred clan formation^29,30^. In general, the presence of offspring plays a major role in social relationships among individuals: families dominate pairs without goslings in aggressive encounters, and pairs tend to win over single birds^31–33^. Greylag geese (*Anser anser*) are long-lived (10 years and longer^34,35^) and lifelong monogamy is the rule (i.e. males and females associate year-round^36^). Outside the breeding season, greylag geese are highly gregarious with strong family bonds; fledged goslings remain with their parents until the next breeding season^36^. Within flocks, agonistic interactions between pairs, families and clans are common. Such agonistic and supportive social contexts strongly modulate both the hypothalamic-pituitary and the sympathetic-adrenergic stress responses^37,38^. The effect of social support depends on family size because, in greylag geese with offspring, the excretion of immune-reactive corticosterone metabolites decreases with the number of offspring^17,38^. Furthermore, social and environmental factors interact with the immune system, as manifest in individual haematology: haematocrit and differential leucocyte counts are contingent with a suite of individual (i.e. sex, age), social (i.e. pair-bond status, parental experience) and environmental factors (i.e. season)^39,40^. Finally, emotional social support, enjoyed by individuals in long-term social bonds, contributes to reducing long-term glucocorticoid levels and thereby may help avoid gastrointestinal diseases related to chronic stress^41^.

We suggest that, via such mechanisms, social context affects the digestive efficiency in the highly social greylag geese, which were a valuable model species for recent social complexity research^42^. In the present study, we investigated the potential link between digestion efficiency and social status in 38 individuals belonging to different social categories (i.e. paired individuals with and without goslings, unpaired ones and juveniles) of a semi-feral and individually marked flock of greylag geese in winter.

The study was conducted during 9 consecutive days in winter 2017, when the ground was fully covered with snow, i.e. no grassland accessible. This enabled us to standardize the quality of food available to the geese. The apparent digestibility of organic matter (ADOM) was assessed from individual droppings by using lignin as an internal marker in food and droppings.

Because of the complex relationships between social context and physiological parameters, we expected paired birds with offspring (i.e. socially well embedded and enjoying social support)^43^ to show a better digestive efficiency than unpaired individuals. Furthermore, we considered ADOM as an indicator of the physiological condition of an individual^44^ at the end of the winter, when the breeding season starts. According to the condition-dependent model, individuals should adjust their reproductive decisions as a function of their body condition^45^. Therefore, we considered ADOM to indirectly modulate the reproductive performance in the following breeding season.

## Results

During the study the birds were fed with standardised pellets with 95.6% dry matter content (DM). Ash and lignin were 7.5% and 5.3%, respectively. The individual droppings collected had an average analytical DM (±SD) of 95.0 ± 1.0%, and a content of 29.3 ± 13.5% and 9.5 ± 2.9% on DM basis for ash and lignin content, respectively. The calculations for lignin and ash were made on DM basis, i.e. on the dry content and not on the total sample (see the methods section below).

**Figure 1:**
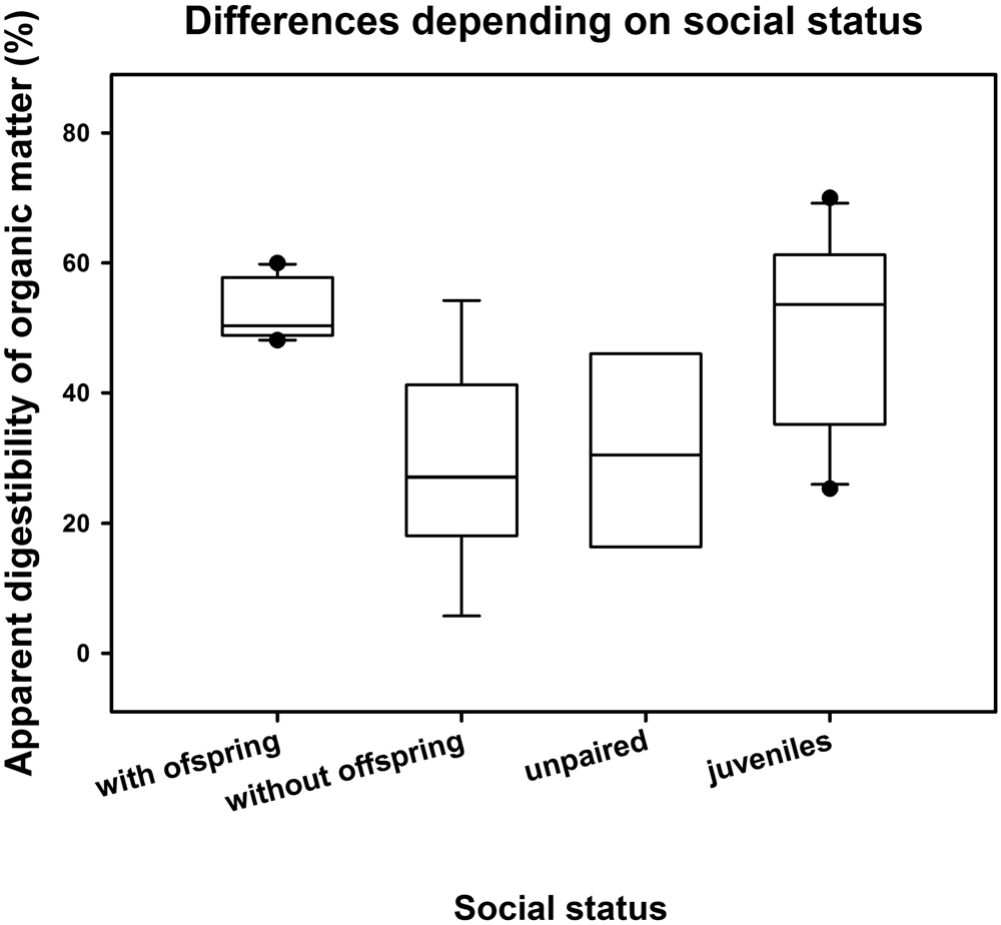
Differences in percentages of ‘apparent digestibility of organic matter’ (ADOM) between different social categories (i.e. paired with and without offspring, unpaired and juvenile individuals). Circles indicate outliers per group; error bars are based on interquartile ranges (lower: 25th and upper: 75th percentile).

Model-averaged results identified social category as the strongest determinant of apparent digestibility of organic matter (ADOM). Paired individuals with offspring showed a significantly higher digestive efficiency than unpaired individuals or paired ones without offspring (Fig. 1). Age was the least important parameter, and sex had no influence on ADOM.

**Figure 2:**
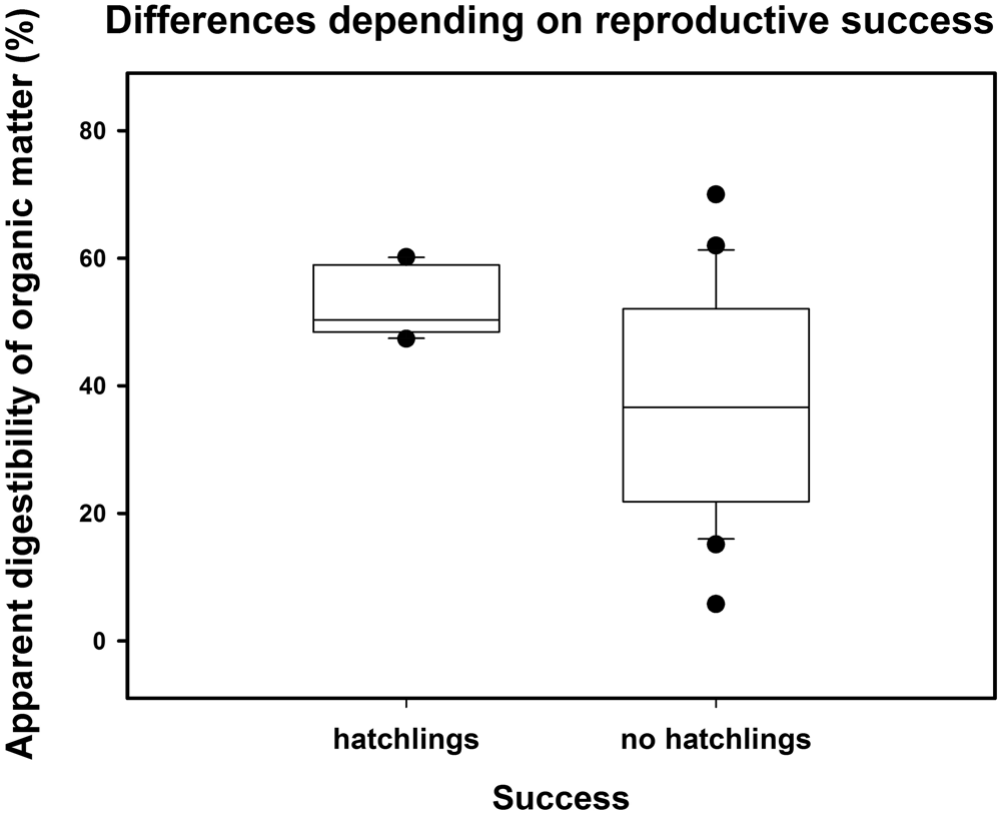
Differences in percentages of ADOM between individuals with goslings and those without goslings in the following season. Circles indicate the outliers per group; error bars are based on interquartile ranges (lower: 25th and upper: 75th percentile).

With respect to the interplay between ADOM in winter and the reproductive success in the following breeding season, hatched goslings was the most influential variable on ADOM. Individuals with hatched offspring in the next season showed a greater ADOM value at the beginning of the mating season than geese without (Fig. 2). Having a nest *per se* and/or fledged goslings had no influence on ADOM.

Top-ranked models are presented in Table 1, model averaged coefficients in Table 2.

**Table 1:** Top-ranked models. Given are the predictors modulating the response variable ‘apparent digestibility of organic matter’ (ADOM).

Response variable	Random factor	Model	df	logLik	ΔAICc	Akaike weight
ADOM	(1|individual)	**Social category**	6	**−153.863**	**0.00**	**0.520**
		**Social category + Age**	7	**−153.118**	**1.53**	**0.242**
		Social category + Sex	7	−153.631	2.56	0.145
		Social category + Age + Sex	8	−152.472	3.47	0.092
		Age	4	−162.705	12.19	0.001
		Age + Sex	5	−162.388	14.21	0.000
		Intercept only	3	−165.046	14.36	0.000
		Sex	4	−165.006	16.79	0.000
ADOM	(1|individual)	**Hatched goslings**	4	**−160.827**	**0.00**	**0.735**
		Hatched goslings + Nest	5	−160.823	2.66	0.195
		Intercept only	3	−165.046	5.93	0.038
		Nest	4	−163.938	6.22	0.033
ADOM	(1|individual)	**Intercept only**	3	**−38.281**	**0.00**	**0.853**
		Fledged goslings	4	−37.873	3.52	0.147

Model selection table. Parameters explaining the response variable apparent digestibility of total organic matter are given. Bold indicates top-ranked models. df – degrees of freedom, logLik – log-likelihood, ΔAICc – differences of the second-order Akaike information criterion between the best model and the other top-ranked models.

**Table 2:** Model-averaged coefficients. Given are the coefficients with adjusted standard errors, lower and upper confidence intervals and relative importance of the top-ranked models.

Response variable	Coefficients	Estimate	Adjusted SE	CI lower limit (2.5%)	CI upper limit (97.5%)	Relative importance
ADOM	Intercept	49.533	4.892	39.945	59.121	
	Social category (paired with offspring)	5.636	8.368	−10.765	22.036	1.00
	Social category (paired without offspring)	−20.931	8.674	−37.931	−3.931	1.00
	Social category (unpaired)	−20.620	8.402	−37.088	−4.152	1.00
	Age	−0.668	0.604	−1.851	0.515	0.32
ADOM	Intercept	52.986	5.491	42.288	63.684	
	Hatched goslings	−18.563	7.435	−33.048	−4.078	1.00

Model-averaged coefficients of the top-ranked models including adjusted standard errors (SE), lower and upper confidence intervals (CI) and relative importance.

For social category, juveniles were set to zero.

## Discussion

Our main findings indicate that social status modulates digestive efficiency, probably by dampening the stress response in socially well-embedded individuals, i.e. paired birds accompanied by their offspring. These results suggest fine tuning mechanisms between social context and physiological parameters as expressed by the percentage of apparent digested organic matter (ADOM). Paired individuals with offspring, irrespective of sex, showed a higher digestive efficiency than paired birds without offspring or unpaired ones. The seasonal patterns of corticosterone in greylag geese discussed by Kotrschal *et al*.^46^ support our findings: early in the mating season, unpaired males excrete more immune-reactive corticosterone metabolites than paired ones. We interpret this as being related to the social situation within the flock because family members enjoy social support and the benefit of lower corticosterone levels compared to lone individuals when the mating season begins^30^. In geese, long-lasting family bonds are known to provide advantages for both the offspring and the parents, as was shown by the high efficiency of digestion in paired individuals with offspring. Juveniles, for instance, benefit in terms of body condition: juvenile barnacle geese (*Branta leucopsis*) within family units were disturbed less during feeding than those that had already left the family unit. Furthermore, Black *et al*.^47^ showed that 10-month-old juvenile barnacle geese in family bonds were in better body condition after migration than those without family bonds. From the parents’ perspective, the accompanying offspring actively contributed to detecting predators and to gaining and defending foraging space. This enabled the parents to spend more time foraging and ultimately enhanced the chances of reproductive success in the immediate future^47,48^. We therefore suggest that a high digestive efficiency linked to social status has a positive impact on the reproductive success of the following season.

Families are generally highest in rank within the social structure of the flock. Experimental studies on barnacle geese suggest that subordinate individuals occupy explorative positions in the front or at the edge, being quickly displaced by dominant geese which tend to monopolize the sites with enriched vegetation. Subordinates compensated for a lower energy intake per time by longer foraging bouts^49^. In our study, however, the provided food was standardised and available *ad libitum*, so that individuals of all social categories were able to forage to satiation.

Juvenile greylag geese generally reach sexual maturity between 1 and 2 years of age. In our study, all focal juveniles were two years old and none of them accompanied the parents, i.e. was involved in a secondary family^30^. Nevertheless, they had a high ADOM, which might reflect different metabolic traits linked with age. However, our analysis indicates age *per se* to be the least important factor affecting ADOM (Table 2). This suggested that other factors are involved, for example metabolic differences linked to attaining sexual maturity and/or to the transition to adulthood, as shown by other studies^50,51^.

Furthermore, ADOM is apparently related to body condition, which is relevant for the forthcoming reproductive performance; focal individuals with hatched offspring in the breeding season following the data collection period showed higher values of ADOM than those failing to hatch goslings. Our study was conducted in February, a few weeks before the beginning of the laying period. ADOM therefore seems to be a meaningful value for an individual’s capability of facing an energetically demanding period such as the egg laying one. However, the correlative characteristic of our approach calls for further experimental study to corroborate such insights.

Additionally, a low digestive efficiency might be compensated by a larger amount of food eaten, which would probably extend foraging time. Thereby, subordinates with low digestive efficiency might be forced to positions within the group that are more exposed to predators^52^, requiring them to make a trade-off between foraging and vigilance behaviour. Saito^53^ claims that in some cases the mere presence of a dominant individual may cause a reduced energetic intake in a subordinate (socially mediated interference)^7^.

We suggest that long-term social bonds go beyond dampening corticosterone levels and benefitting both haematological and immunological parameters^17,18,54^ to also affect further stress-related parameters such as digestive efficiency. In fact, socially well-embedded individuals benefit from better nutrient absorption than individuals lacking the supportive presence of social allies within the flock. Similar patterns have been shown for humans and non-human primates^55^. Such benefits might be mediated by increased levels of mesotocin, the analogue of mammalian oxytocin in birds, which facilitates the release of simpathethic-controlled gastrointestinal hormones and weight gain^56,57^. In mammals, oxytocin plays a major role in regulating social behaviour and positive interactions, thereby facilitating bonding or attachment by increasing social contact between individuals^58–61^. Babygirija *et al*.^59^, for instance, showed that social attachment allows rats to overcome daily stressful events and improve the impaired gastric motor function by up-regulating central oxytocin expression.

Our study provides new insight into the effects of complex social life on behavioural physiology of a model bird species and suggests further experimental research to understand the relationship between ADOM and life history traits.

## Material and Methods

### Study area and focal animals

The study area is located at 550 m above sea level in the valley of the Alm River in the northern part of the Austrian Alps (47°48**′**E, 13°56**′**N). The non-migratory flock of greylag geese was introduced by K. Lorenz and co-workers in 1973^62^. The birds are unrestrained and suffer natural predation with losses of up to 10% of the flock per year^35^. The geese are individually marked with coloured leg rings and are habituated to the close presence of humans^37,38^. Individual life-history data have been collected since 1973, which provides reliable information about an individual’s social relationships within the flock (i.e. paired or not) as well as information on reproductive performance (i.e. having a nest or not; having hatched/fledged goslings or not). During the period of data collection, the flock totalled 167 individuals. Focal birds were 38 individuals (20 males and 18 females) belonging to different social categories within the flock, i.e. paired with and without offspring, unpaired individuals and juveniles (5 males and 3 to 5 females per category, Table 3). Age ranged from 2 to 24 years (mean age ± SD = 9.86 ± 6.43).

**Table 3:** List of the focal individuals, their social status at the time of data collection, their age (years), sex (m = male, f = female) and information about their reproductive performance.

Goose	Social category	Nr.droppings	Sex	Age	Nest?	Goslings?
JB	pairedwithoffspring	4	m	17	yes	yes
Allegra	pairedwithoffspring	5	m	17	yes	yes
Lionel	pairedwithoffspring	5	m	8	yes	yes
Kordula	pairedwithoffspring	5	m	8	yes	yes
Cap	pairedwithoffspring	5	m	12	yes	no
Leviathan	pairedwithoffspring	5	f	16	yes	yes
Duftspur	pairedwithoffspring	5	f	16	yes	no
Lowenherz	pairedwithoffspring	4	f	16	yes	yes
Luke Skywalker	pairedwithoffspring	6	f	8	yes	yes
Erna	pairedwithoffspring	5	f	8	yes	yes
Iwan	pairedwithoutoffspring	5	m	24	no	no
Tipi	pairedwithoutoffspring	5	m	18	no	no
Halas	pairedwithoutoffspring	4	m	17	yes	no
Terri	pairedwithoutoffspring	5	m	17	yes	no
Bayou	pairedwithoutoffspring	5	m	10	yes	no
Lestate	pairedwithoutoffspring	5	f	12	no	no
Ginny	pairedwithoutoffspring	5	f	11	yes	no
Pippin	pairedwithoutoffspring	5	f	10	no	no
Luna	pairedwithoutoffspring	5	f	9	yes	no
Lilla	pairedwithoutoffspring	4	f	9	yes	no
Bailey	unpaired	5	m	17	no	no
Smoky	unpaired	5	m	16	no	no
Bilbo	unpaired	5	m	14	no	no
Medea	unpaired	5	m	8	no	no
Judith	unpaired	5	f	19	no	no
Bonsai	unpaired	5	f	4	no	no
Lenka	unpaired	4	f	4	yes	no
Kirgistan	juvenile	4	m	2	yes	no
Leberblumchen	juvenile	5	m	2	no	no
Leonidas	juvenile	5	m	2	yes	no
Babaco	juvenile	4	m	2	yes	yes
Glen Grant	juvenile	5	m	2	no	no
Kamerun	juvenile	5	f	2	no	no
Lavender	juvenile	5	f	2	yes	no
Banane	juvenile	5	f	2	no	no
Dorothea	juvenile	5	f	2	yes	yes
Diamante	juvenile	5	f	2	no	no

### Data collection

Individual droppings were collected during 9 consecutive days in winter 2017, from 14 to 22 February. During this period the snowpack was completely closed (i.e. no grassland was available), which ensured standardisation of the foraging situation: the birds fed exclusively on the provided pellets, which were available *ad libitum*. To our knowledge, there is no evidence for diurnal variation of digestive efficiency. Therefore, samples were collected twice a day, starting approximately one hour after feeding of the birds and lasting for 2.5 hours, i.e. between 0900–1130 and 1600–1800.

Droppings were collected immediately after defecation in 1 l plastic bags – one sample per bag – and frozen at −20 °C within 1 h until further analysis. A total number of 184 individual droppings were sampled from the focal birds (mean number of droppings per individual ± SD = 4.8 ± 0.4). After data collection was completed, samples were dried in an oven for 24 h at 55 °C and milled through a 1 mm screen (Pulverisette; Fritsch, Idar-Oberstein, Germany). Both the provided pellets and the collected droppings were analysed for (i) dry matter (DM) content by heating at 105 °C for 3 h (method 930.15^63^), (ii) ash by incineration at 550 °C for 2 h (method 942.05^63^) and (iii) acid detergent lignin^64^. Lignin was used as an internal marker to calculate the apparent digestibility of organic matter (ADOM) according to the following formula^65^: apparent digestibility (%) = [(1 − lignin in pellets) (g)/lignin in droppings (g)] × 100.

### Statistical analysis

Data were processed by using R version 3.2.5^66^ and the additional packages ‘nlme’^67^ for linear mixed effects models (LME), and ‘MuMIn’ (Multi-Model Inference^68^); for information-theoretic model selection and model averaging based on information criteria.

The distribution of the residuals was analysed with Shapiro-Wilk tests and visual inspection of Q-Q-Plots. To assess different effects on ADOM (response variable), we used LMEs(type-III sum of squares) with either (1) social category (paired with or without offspring, unpaired and juvenile), sex and age (years), (2) reproductive success of the following season (nest: yes/no; hatched goslings: yes/no) or (3) fledged goslings of the following breeding season (yes/no; sub dataset: including only pairs with at least one hatched offspring) as fixed factors. Bird identities were included as random factors in all models to control for the repeated sampling from individual birds. Three different regression models were used because fixed factors with multiple levels were included in the first model. To avoid overfitting and successfully calculate the models, a second model with the same dataset was used. The third model contained a sub-dataset of the original one for the parameter “fledged goslings”.

To select the best models, an information-theoretic approach was used to calculate all possible candidate models^69^. All full models were compared to their corresponding null model. We ranked them according to their second-order form of Akaike’s information criterion (AICc to account for small sample sizes^70^), and selected the top-ranked models with a ΔAICc ≤ 2. The models were averaged to create model-averaged coefficients^69^. Those models in which the variation of the explanatory variables was not better explained by the full model than the null model (i.e. both models ΔAICc ≤ 2) are not presented in Table 2.

### Ethical statement

This study complies with all current Austrian laws and regulations concerning working with wildlife. All data were collected non-invasively under Animal Experiment Licence Nr. BMWF-66.006/0011-WF/II/3b/2014 by the Austrian Federal Ministry for Science and Research.The authors adhere to the ‘Guidelines for the use of animals in research’ as published in Animal Behaviour (1991, 41, 183–186).

## Electronic supplementary material

Supplementary Dataset 1

## Data Availability

Data are provided in the electronic supplementary material appendix S3.
